# Regulation of SUMOylation Targets Associated With Wnt/β-Catenin Pathway

**DOI:** 10.3389/fonc.2022.943683

**Published:** 2022-06-30

**Authors:** Linlin Fan, Xudong Yang, Minying Zheng, Xiaohui Yang, Yidi Ning, Ming Gao, Shiwu Zhang

**Affiliations:** ^1^ Graduate School, Tianjin University of Traditional Chinese Medicine, Tianjin, China; ^2^ Tianjin Rehabilitation Center, Tianjin, China; ^3^ Department of Pathology, Tianjin Union Medical Center, Tianjin, China; ^4^ Nankai University School of Medicine, Nankai University, Tianjin, China; ^5^ Department of Thyroid Surgery, Tianjin Union Medical Center, Tianjin, China

**Keywords:** sumoylation, Wnt, β-catenin, t-cell factor, lymphoid enhancer factor

## Abstract

Wnt/β-catenin signaling is a delicate and complex signal transduction pathway mediated by multiple signaling molecules, which plays a significant role in regulating human physiology and pathology. Abnormally activated Wnt/β-catenin signaling pathway plays a crucial role in promoting malignant tumor occurrence, development, recurrence, and metastasis, particularly in cancer stem cells. Studies have shown that the Wnt/β-catenin signaling pathway controls cell fate and function through the transcriptional and post-translational regulation of omics networks. Therefore, precise regulation of Wnt/β-catenin signaling as a cancer-targeting strategy may contribute to the treatment of some malignancies. SUMOylation is a post-translational modification of proteins that has been found to play a major role in the Wnt/β-catenin signaling pathway. Here, we review the complex regulation of Wnt/β-catenin signaling by SUMOylation and discuss the potential targets of SUMOylation therapy.

## Introduction

The wingless and Int-1 (Wnt)/β-catenin signaling pathway is a key cascade in embryonic development, self-renewal of organisms, cell proliferation, differentiation (1–3), and usually involves in various cancers, such as colorectal cancer (4), breast cancer (5), gastric cancer (6), ovarian cancer (7), pancreatic cancer (8), prostate cancer (9), leukemia (10), and melanoma (11). Abnormal activation of this pathway plays an essential role in chemoradiotherapy resistance (12). In addition, Wnt/β-catenin has emerged as a critical regulator of cancer stem cells (CSCs), which is considered one of the root causes of cancer recurrence and metastasis because of their heterogeneity and plasticity (13). Furthermore, the Wnt/β-catenin can crosstalk with other signaling pathways including Notch, FGF, Hedgehog, and TGF-β/BMP signaling cascades to form a signaling network to regulate the survival and progression of cancer cells (14–16). Post-translational modifications (PTMs) of proteins, including phosphorylation, acetylation, ubiquitination, and SUMOylation, can regulate the function of proteins, determine the active state and subcellular location of proteins, and dynamically interact with other proteins related to carcinogenesis and progression (17–20). Conceptually, it is an efficient way to treat cancer involved the hijacking of PTMs of the key molecules in Wnt/β-catenin. SUMOylation of proteins is an important mechanism in cellular responses to environmental stress (21, 22). Recent reports based on proteomic studies have identified many SUMOylated substrates that play important roles in the development and progression of cancer. Proteins associated with the Wnt/β-catenin pathway have been identified as SUMOylated substrates, and evidences suggested that the initiation and progression of cancers depended on the function of the SUMOylation (23). This suggests the possibility that strictly regulated self-renewal mediated by Wnt signaling in cancer cells may be disturbed by the SUMOylation pathway to allow more malignant proliferation. Hence, some studies have indicated that targeting the SUMOylation modification in Wnt/β-catenin might be a strategy for the treatment cancer (24).

Following the first member of the Wnt family identified over the last four decades (25), several recent studies have focused on the Wnt/β-catenin signaling pathway (26, 27). This has led to an improved understanding of the multilayered mechanisms of this pathway transduction proceeds as well as the molecular mechanism regulating this pathway (28). Signaling is initiated when the Wnt ligand binds to the Frizzled receptor on the cell membrane and the LDL receptor-associated protein 5/6 (LRP5/6) co-receptor. This receptor can induce phosphorylation of dishevelment (DVL) and recruitment of Axam to destroy the destructive complex (29). This destructive complex include adenomatous polyposis coli (APC), glycogen synthase kinase 3β (GSK-3β), Axin, and casein kinase 1 (CK1), which promote ubiquitination-dependent degradation of phosphorylated β-catenin (30). Dephosphorylated β-catenin is then translocated to the nucleus where it initiates intracellular signal transduction. In the nucleus, β-catenin can bind to members of the transcription factor (T-cell factor/lymphoid enhancer factor TCF/LEF) family and recruit transcription coactivators and CREB binding protein (CBP) to the transcription of the Wnt target genes (31–33). β-catenin in the nucleus plays key regulatory roles in cancer cell target genes, including c-Myc (34), CD44 (35), Lgr5 (25, 36), and axis inhibition protein 2 (Axin2) (37). The C-terminal binding protein (CtBP) can bind to various transcription factors and play a bidirectional regulatory role for the transcription of Wnt target gene (38, 39).

SUMOylation of proteins is a process in which small ubiquitin-like modifier (SUMO) proteins bind to substrates covalently or non-covalently under the catalysis of SUMO proteases (40). SUMO proteins in mammalian cells present four SUMO isoforms: SUMO1, SUMO2/3, and SUMO4 (41). Although SUMO2 and SUMO3 cannot be distinguished by antibodies as they are very similar, SUMO1 is quite divergent. Furthermore, the expression levels of SUMO2/3 exceed SUMO1 in human cells (42). The SUMO4 precursor possess proline at amino acid 90, which seems to limit its processing to yield a mature form (43). Despite this difference, all SUMO proteins are activated and conjugated by the same SUMO proteases in an orderly manner. SUMO proteases are composed of three types of enzymes: SUMO-activating enzymes (E1), SUMO-conjugating enzymes (E2), and SUMO-specific ligases (E3) (44). E1s are composed of two subunits: SAE1/Aos1 and SAE2/Uba2 (45). There is only one E2, UBC9, among mammals (46). With the advancement of SUMOylation research, an increasing number of E3s have been identified, including the PIAS family (PIAS1, PIASx, PIAS3, and PIASy), RanBP2, and the human polycomb 2 (Pc2) (47, 48). The SUMO enzymes and proteins are predominantly enriched in the nucleus. However, they have also been detected in other subcellular locations (cytoplasm, Golgi apparatus, and plasma membrane) (49). Three distinct but not mutually exclusive mechanisms determine whether proteins can be SUMOylated (40). Firstly, substrates can directly interact with Ubc9 (SUMO consensus site-directed SUMOylation). Secondly, the substrate possesses the SUMO interaction motif (SIM) (SIM-dependent SUMOylation). Thirdly, proteins are recognized by a SUMO E3 ligase, which is accessible to Ubc9 (E3 ligase-dependent SUMOylation). Although all SUMO isoforms use the same enzymatic mechanism, the underlying mechanism determining their specificity remains unclear. SUMOylation of proteins is a multi-step enzymatic cascade reaction in which the first step of the SUMOylation modification is the maturation of SUMO proteins by SUMO-specific proteases (50). Mature SUMO proteins are then directly activated by SUMO E1 *via* ATP hydrolysis. Consequently, upon interaction of the charged E1 enzyme with E2, SUMO is transferred from E1 to E2, forming an E2-SUMO thioester. Finally, SUMO is linked to the lysine residues of target proteins *via* an isopeptide bond, with or without stimulation by the ligase enzyme (E3). SUMOylation of proteins provide a platform for enhancing protein-protein interactions (51, 52).

DeSUMOylation is the process of removing the SUMO from SUMOylated substrates (53, 54). The proteins that perform the function of removing SUMO are mainly two different enzymes: SUMO-specific proteases and SUMO-targeted ubiquitin ligases (STUbLs) (44). The main function of SUMO-specific proteases is to promote maturation of and activate the SUMO protein. DeSUMOylation can also remove SUMO protein from the substrate protein and inhibit SUMOylation of the substrates. The bidirectional action of the enzyme may depend on the output of the integrated signal in the environment in which the enzyme is located. With the discovery of sentrin-specific protease 1 (SENP1) (55), more SUMO-specific proteases have been discovered, including SENP1-SENP3 and SENP5-SENP7 (56). Subsequently, three novel SUMO-specific proteases have been identified in humans: desulphurized isopeptidase 1 (DeSI1), desulphurized isopeptidase 2 (DeSI2), and ubiquitin-specific protease-like 1(USPL1) (57, 58). Compared to the SENP protease, the three novel SUMO-specific proteases have a unique binding sequence pattern. In addition, STUbls, a family of functionally conserved enzymes, can negatively regulate SUMOylation levels. They catalyze the addition of ubiquitin to proteins previously modified by SUMOylation and promote protein degradation, illustrating that SUMO can indirectly promote protein degradation, such as RNF4 and Arkadia (RNF111) (59). The sub-cellular localization and activity of these enzymes in response to different stimuli are effective mechanisms to adjust cellular SUMOylation levels (60, 61).

**Figure 1:**
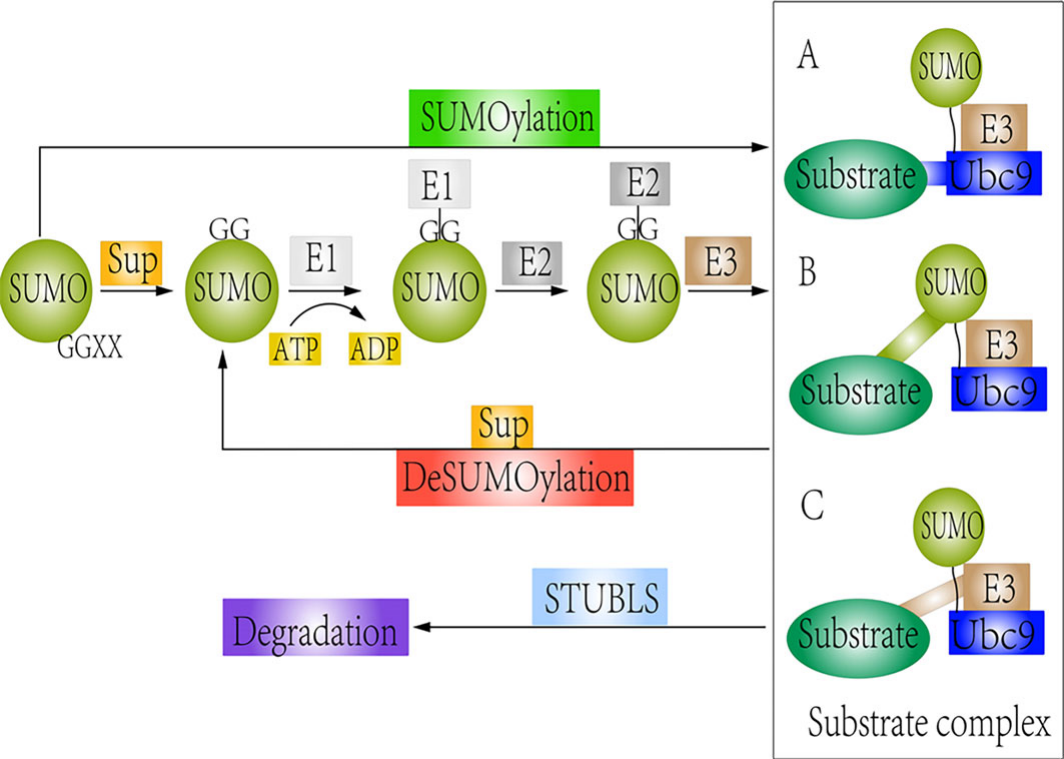
Schematic representation of SUMOylation and deSUMOylation. The first step is maturation of the SUMO protein. Then, mature SUMO is directly activated by SUMO E1 *via* ATP hydrolysis. Upon interaction of the charged E1 enzyme with E2, SUMO is transferred from E1 to E2. Finally, SUMO links to substrates by different forms: **(A)** Substrates directly interact with the Ubc9 (SUMO consensus site–directed SUMOylation); **(B)** Substrates possess the SUMO interaction motif (SIM) (SIM-dependent SUMOylation). **(C)** Proteins are recognized by a SUMO E3 ligase, which can be accessible to the charged Ubc9 (E3 ligase-dependent SUMOylation). However, SUMO-specific proteases could remove the SUMO protein from the substrate. STUbls promote degradation of SUMOylated substrate.

SUMOylation and deSUMOylation remain in a dynamic equilibrium, allowing cells to respond rapidly to changing external and internal pressures and stimuli (44, 62). SUMOylation and deSUMOylation are involved in cellular processes such as metabolism, DNA repair, and signal transduction (40, 63). Dysregulation of SUMOylation and deSUMOylation can lead to severe defects in cell proliferation and genomic stability. In this review, we systematically describe the crosstalk between Wnt/β-catenin signaling and the SUMOylation modification and briefly emphasize its therapeutic potential for treating cancer. The detail regulatory mechanisms between SUMOylation and deSUMOylation are shown in **Figure 1**.

## SUMOylation is Essential for Nuclear Import of β-Catenin

β-catenin is an evolutionarily conserved molecule that plays crucial roles in a variety of developmental and homeostatic processes (64). Aberrant activation of β-catenin is associated with cancer. More specifically, β-catenin is not only a structural component of the adhesion junctions of cadherins, but also a key nuclear effector of Wnt/β-catenin signaling (64). The Wnt/β-catenin pathway is ultimately centered on the PTM of β-catenin to maintain its abundance and regulate its subcellular localization (28). Accumulated β-catenin can translocate from the cytoplasm to the nucleus and participate in the transcription of multiple target genes. Nuclear translocation of β-catenin is regulated by multiple PTMs, including phosphorylation, acetylation, ubiquitination, and SUMOylation (65–68). However, several studies demonstrated that β-catenin had no recognizable nuclear localization signal (NLS) that could be transported to the nucleus by SUMOylation (67). To date, the mechanism of the interaction between β-catenin and the SUMOylation modification is not completely clear. All the studies mentioned above are based on interactions with other proteins, and there are no reports on whether β-catenin itself has SUMOylated modification sites to bind SUMO.

RanBP2 is part of the nuclear pore complex (NPC) and acts as a SUMO E3 enzyme, which helps macromolecular proteins that lack nuclear localization signals to enter the nucleus (69). It promotes the formation of the TCF-4-SUMO1-RanGAP1-Ubc9-RanBP2 complex by enhancing the SUMOylation of TCF-4 and the hydrolysis of RanGTP by RanGAP1, which binds to β-catenin and promotes its nuclear translocation (70). Another study demonstrated that total β-catenin and nuclear β-catenin expression were significantly decreased and phosphorylated β-catenin expression was increased after Uba2, an important component of the E1 activating enzyme, was knocked out (71). Briefly, β-catenin is able to enter the nucleus through SUMOylation of interacting proteins. However, the phosphorylation level of β-catenin in the cytoplasm increases when SUMOylation is inhibited, which promotes ubiquitination-dependent degradation of β-catenin. The SUMOylated modification appears to be the “ON/OFF” switch that regulates the nuclear import of β-catenin.

Jiang et al. demonstrated that the deSUMOylation activity of SENP2 was important for β-catenin stability. SENP2, a deSUMOylation enzyme, was downregulated in hepatocellular carcinoma (HCC) tissues (72). The study found that the stability of β-catenin was significantly reduced, whereas overexpression of SENP2 inhibited the growth and colony formation of HCC cells (72). Choi et al. found that SUMOylation excluded TBL1-TBLR1 from the nuclear hormone receptor inhibitor (NCoR) complex and increased recruitment of the TBL1-TBLR1/β-catenin complex to the Wnt target gene promoter, resulting in the expression of the Wnt signal target gene. In contrast, SENP1 reduces the formation of the TBL1-TBLR1/β-catenin complex, resulting in the inhibition of β-catenin-mediated transcription (73).

These studies suggest that the dynamic balance between SUMOylation and deSUMOylation can maintain the stability and transcriptional activity of β-catenin. The TCF-4-SUMO1-RanGAP1-Ubc9-RanBP2 complex can be used as a target to prevent the SUMO modification of β-catenin. β-catenin without SUMO modification cannot enter into the nuclear and play the role in the initiation and development of cancer.

## SUMOylation is a Vital Regulator of Kinase Activity, Protein Stability, and Nuclear Localization of GSK-3β

GSK-3β is a ubiquitously expressed, broadly specific, serine/threonine kinase. Therefore, GSK-3β regulates a number of intracellular signaling pathways and is involved in a variety of biological events, including autophagy, cell apoptosis, and cancer progression (74–77). This is the axis of the classical Wnt signaling pathway (78). GSK-3β promotes β-catenin phosphorylation, leading to ubiquitin-mediated proteolysis. Active GSK-3β is considered a cancer suppressor as it promotes the destruction of several oncogenic proteins [e.g., β-catenin, c-Myc, and myeloid cell leukemia sequence 1 (MCL-1)] (79). Moreover, GSK-3β has also been found to exhibit carcinogenic properties, as it up-regulates pathways critical for cancer cell survival and drug resistance (80).

The SUMOylation of GSK-3β can occur *in vitro* or *in vivo* and is critical for GSK-3β function (81). GSK-3β has been identified as a potential SUMO substrate in proteomic studies, and the lysine residue K292 within the activation ring of GSK-3β has been identified as a SUMO receptor site. SUMOylation is a positive regulator of kinase activity, protein stability, and nuclear localization of GSK-3β. In addition, mutation of the SUMOylation sites in GSK-3β reduces its kinase activity and stability, significantly increasing cell survival (81). L. Eun Jeoung, H, et al. evaluated that the effects of GSK-3β SUMOylation on β-catenin stability and Wnt signaling and confirmed that GSK-3β SUMO mutant (K292R) increased the cell survival rate compared to the wild type GSK-3β (81). However, direct evidence is not sufficient to prove this. Wetzel et al. identified the lysine residue K730 in human ZO-2 as a potential SUMO-modification site directly bound to SUMO1 (82). SUMOylated ZO-2 binds directly to GSK-3β and promotes the kinase activity of GSK-3β. It is inhibited in the presence of SENP1, but not by inactivated SENP1 protein (82). SUMOylation of GSK-3β and upregulation of specific SUMO E3 enzyme of GSK-3β in the tumor microenvironment can inhibit the growth of malignant tumor, which may be used as a potential target for cancer treatment.

## Activation of TCF/LEF Could be Regulated by SUMOylation

Constitutive activation of TCF/LEF is a key downstream effector of the Wnt/β-catenin pathway and is often observed in lung, breast, and colorectal cancers (83). The human TCF/LEF family consists of four homologous members: TCF-1, LEF-1, TCF-3, and TCF-4 (84). TCF-4 is found to be conjugated to SUMO at an endogenous level (70). The SUMOylation site of TCF-4 is Lys297. When Lys297 is mutated to arginine, activation of TCF-4 is inhibited. SUMOylation of TCF-4 can be amplified by PIASy, a SUMO E3 enzyme, and suppressed by Axam, a deSUMOylation enzyme (70). PIASy enhances TCF-4/β-catenin-dependent transcriptional activity, whereas Axam inhibits the SUMOylation of TCF-4. Conversely, lowering Axam protein levels by siRNA resulted in increased SUMOylation of TCF-4 and activation of TCF-4 (70). These results suggest that SUMOylation of TCF-4 is involved in β-catenin-dependent and TCF-4-mediated gene expression in the Wnt signaling. LEF1 is a transcription factor and can activate β-catenin-dependent transcription (85). S. Sachdev, L. et al. identified that LEF1 could be covalent modified by SUMO at Lys25 and Lys 267 (86). PIASy, as a novel interaction partner of LEF1, could significantly increase SUMOylation of LEF1. However, LEF1 activity is effectively inhibited after co-expression of PIASy with LEF1, suggesting that SUMOylated LEF1 inhibits the activation of Wnt/β-catenin pathway (86).

## SUMOylation of Axin has No Effect on β-catenin Rather Than JNK

Axin is a multi-domain protein that interacts with several other proteins and acts as a negative regulator of Wnt signaling by down-regulating β-catenin levels (87, 88). Axin is found to interact with SUMO1 and three SUMO E3 ligase enzymes, PIAS1, PIASx-β, and PIASy (89). Six amino acids in the C-terminus of Axin interact with SUMO. The mutation of the six amino acid residues significantly reduces the expression of SUMOylated Axin (89). Both the mutation-type and wild-type Axin can decrease the transcriptional activity of LEF1 and destabilize β-catenin. SUMOylation of Axin has no effect on Wnt/β-catenin signaling. In contrast, removing any SUMO sites on Axin could reduce JNK activation (89). This indicates that SUMOylation of the same protein will have different effects on different interacting proteins, which depends on the subcellular location of the protein and the crosstalk between other signaling pathways. SUMOylation modification of Axin can effectively cause apoptosis *via* the JNK pathway rather than the Wnt/β**-**catenin pathway.

## Axam and DeSUMOylation

Axam (also called SENP2) belongs to the ubiquitin-like protease 1 (Ulp1) cysteine protease family, which possesses hydrolase and isopeptidase activities of the SUMO-specific proteases (90). Axam usually localizes to the nucleoplasmic side of NPC by non-covalent binding with SUMO (91). A recent study showed that Axam was down-regulated in bladder cancer cells and HCC tissues (92). Additionally, Axam is identified as a novel Axin-binding protein that inhibits Wnt signaling by inhibiting the binding of DVL to Axin (93). Axam loses its deSUMOylation activity when its catalytic domain is mutated, which restores β-catenin activity. These results indicate that Axam acts as a deSUMOylation enzyme and is involved in negative regulation of the Wnt signaling pathway (94). Another study identified a new SUMO-specific protease called XSENP1 in *Xenopus laevis* that inhibited the expression of Wnt signal transduction target genes in a manner similar to that of Axam (95). Usually, Axam cannot be used as an independent therapeutic target for cancer treatment because of its non-specific deSUMOylation activity.

## The Self-Acetylation and SUMOylation of CBP Compete With Each Other

CBP is a histone acetyl transferase and transcription cofactor that regulates the expression of genes involved in a wide range of cellular processes (96). CBP has attracted great interest as a promising new epigenetic target for a variety of diseases, including malignancies, since its discovery in 2006 (97). Studies showed that CBP could be covalently modified by SUMO1 *in vitro* and *in vivo* (98, 99). This covalent modification occurs at lysine residues 999, 1034, and 1057 of the CBP protein. CBP SUMOylation can be reversed by the overexpression of SENP2, a SUMO-specific protease (98). SUMO modification negatively regulates CBP transcriptional activity by recruiting the death domain-associated protein (Daxx) (98). Because CBP does not bind to DNA on its own, SUMO modification inhibits the intrinsic transactivation of CBP, and decreases the activity of Wnt signaling pathway. SUMO1 binding the ZZ domain in CBP is identified using nuclear magnetic resonance (NMR) spectroscopy (100). This SUMO site represents a unique SUMO interaction epitope that is spatially opposed to the epitope observed in a typical SIM. The presence of the ZZ domain in CBP enhances SUMOylation (100). Another study showed that the bromodomain (BRD) and plant homeodomain (PHD) domains of CBP, in addition to the ZZ domain, were the sites of interaction between SUMO1 and Ubc9, further suggesting that CBP might act as a SUMO E3 ligase enzyme to promote intramolecular SUMOylation of adjacent cycle regulatory domain 1 (CRD1) (99). The competition between self-acetylation and SUMOylation of lysine residues in CRD1 determines that CBP responds to various signals as a transcriptional activator or as a suppressor (99). The roles of the different modifications (self-acetylation and SUMOylation) of CBP in Wnt/β**-**catenin pathway need to clarify in the future.

## SUMOylation of CtBP1 Profoundly Affects its Subcellular Localization and Transcriptional Activity

Mammalian CtBPs (CtBP1 and CtBP2) bind to various transcription factors and play a bidirectional regulatory role as a gene-specific activator and repressor of Wnt target gene transcription (39, 101). In earlier investigation, CtBPs played its role in transcriptional repression (102). However, CtBP can directly co-activate TCF4/LEF at key sites of the target genes to promote CSC self-renewal in CRCs (38). Lin et al. reported that SUMOylation of CtBP1 occurred at a single lysine residue (Lys428) of CtBP1, which was promoted by PIAS1 and PIASx-β. SUMOylation of CtBP1 substantially affected its subcellular localization and transcriptional activity (103). CtBP1 is transferred from the nucleus to the cytoplasm and loses its transcriptional activity after mutation at the SUMOylated Lys428 site (103, 104). Pc2 is a SUMO E3 ligase enzyme and significantly amplifies CtBP SUMOylation (105–107). Riefler et al. demonstrated an increase in nuclear accumulation of CtBP1 after binding to SUMO (108). The decreased SUMOylation of CtBP1 can result in the cytoplasmic retention of CtBP1 (108). These studies suggest that SUMOylation plays a critical role in nuclear retention of CtBP and positively regulating the activity of Wnt signaling pathway. Notably, CtBP2, a close homolog of CtBP1, lacks SUMOylation sites and is not modified by SUMO1. In addition, SUMOylation of friend of GATA1 could promote the interaction with members of the CtBP family, particularly CtBP1 (109).

## SUMOylation of Smad3/4

Wnt/β-catenin and Smad3/4 can crosstalk with each other (110). In the cytoplasm, Smad3 directly promotes the nuclear translocation of β-catenin (111), which regulates the expression of different target genes. Several studies have shown that inactivation of Smad4 can promote self-renewal of CSCs (112). As a cancer suppressor, Smad4 is involved in Wnt-Kras-mediated inhibition of colorectal cancer. The loss of Smad4 and activation of Wnt signaling can create conditions favorable to the fate of stem cells and enable them to re-enter the cell cycle (113). Freeman et al. demonstrated that reduced levels of Smad4 correlated with increased levels of β-catenin mRNA (114). Smad3/4 is a target of PTMs, such as phosphorylation, ubiquitination, SUMOylation, and ADP ribosylation. However, the effect of PTM of Smad3/4 on Wnt signaling, particularly SUMOylation, has been poorly reported.

Recent data indicated that SUMO1 could bind to the C-terminal domain of Smad3, which was increased in the presence of PIASy, resulting in its translocation to the cytoplasm *in vivo* and *in vitro* (115). The cytoplasmic Smad3 can promote the nuclear translocation of β-catenin and activate Wnt signaling pathway. The co-expression of PIASy and SUMO1 not only affects the subcellular localization of Smad3 but also affects the binding activity of Smad3 to DNA. PIASy can regulate TGF-β/Smad3-mediated signaling by stimulating SUMOylation and nuclear output of Smad3 (116). SUMO1 and SUMO2/3 could bind to Smad4 *in vitro* and *in vivo* (117, 118). SUMOylation affects the stability, transcriptional activity, and subcellular localization of Smad4 (119). The major SUMOylation site in Smad4 is located in its linker fragment at Lys159, with an additional site at Lys113 in the MH-1 domain. Double mutations at these two sites enhance Smad4 stability (120). SUMOylation of Smad4 is amplified by PIAS1, PIASx-α, PIASx-β, and PIASy (118). Overexpression of SUMO1 and Ubc9 inhibits TGF-β reactivity reporter genes, whereas co-transfection with SUMO1 protease-1 (SuPr-1) increases TGF-β recovery (117). In addition, direct fusion of Smad4 with mutant sites (K113R and K159R) and SUMO1 effectively inhibits its transcriptional activity (117). Lee et al. reported that Smad4 could be modified by SUMO2/3 (but not SUMO1) to further activate TGF-β/Smad signaling in mesangial cells under high glucose conditions (121). Liu et al. demonstrated that ginkgolic acid (GA), a SUMO1 inhibitor, could inhibit carcinogenesis and cancer progression in oral squamous cell carcinoma by inhibiting Smad4 SUMOylation in a time- and concentration-dependent manner (122). The transcriptional activity of SUMOylationed Smad4 can be stimulated or inhibited in different cells at different niches, which may lead to different transcriptional effects.

## CD44 and SUMOylation

**Figure 2:**
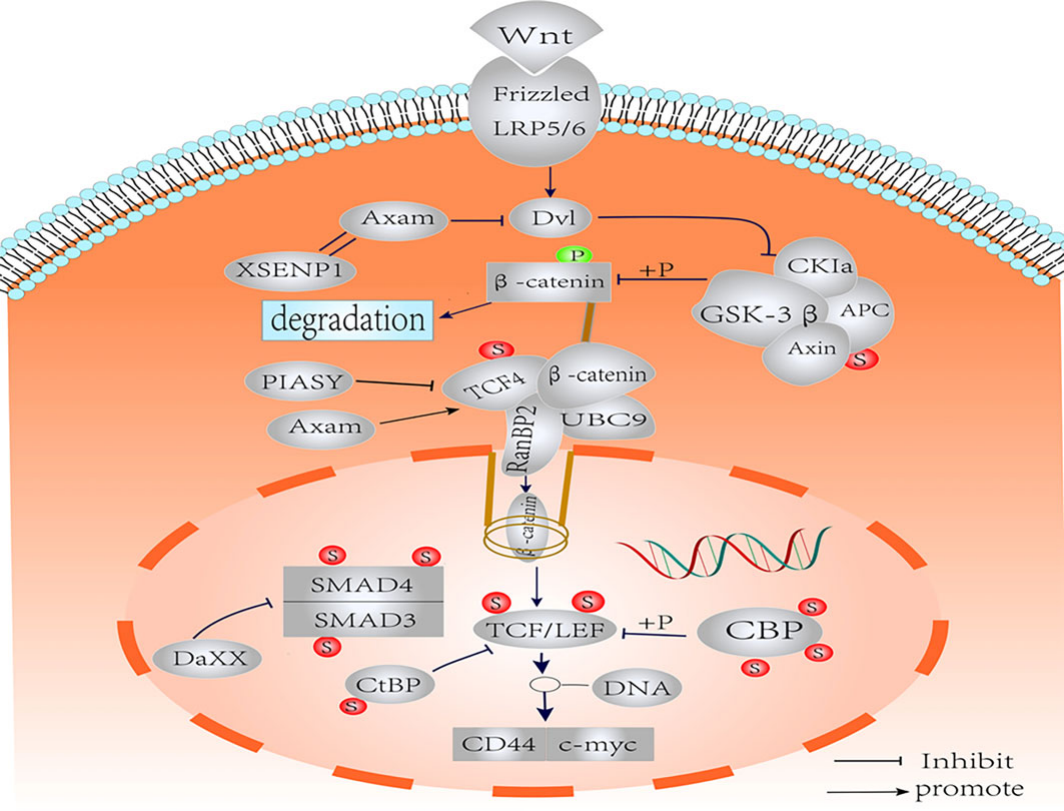
Schematic representation of the SUMOylation on the components of Wnt/β-catenin pathways. Nuclear translocation of β-catenin is regulated by SUMO. The SUMOylation of TCF-4 increases the activity of TCF-4. SUMOylated TCF promotes the binding of β-catenin to RanBP2 of the nuclear pore complex into the nucleus. SUMOylation of GSK-3β improves kinase activity and protein stability. The SUMOylation of LEF1 and its co-expression with PIASy resulted in effective inhibition of LEF1 activity. The SUMOylation of Axin had no effect on Wnt signaling. Axam acts as deSUMOylated enzyme to downregulate β-catenin and implicates the negative regulation of Wnt signaling pathway. SUMO modification negatively regulates CBP transcriptional activity by recruiting Daxx. The CBP may act as the SUMO E3 ligase enzyme, which promotes intramolecular SUMOylation of adjacent CRD1 domains. Binding of CtBP1 to SUMO increases nuclear accumulation. Smad3 SUMOylation is enhanced in the presence of PIASy, resulting in translocation to the cytoplasm. Under the influence of PIASy, PIAS1, PIASxα, and PIASxβ, SUMOylation reduces the stability and transcriptional activity of Smad4, but the effect on Wnt/β-catenin is not clear.

CD44 is a member of the transmembrane glycoprotein family (123), and involves in several physiological and pathological processes, including hematopoiesis, inflammation, and cancer (124, 125). CD44, as a CSC marker, is a key target gene of the Wnt signaling pathway (35, 126), and can positively regulate Wnt signaling by regulating the localization and activation of LRP6 (127). Although few studies have focused on the PTM of CD44, it has been confirmed that CD44 was regulated by SUMOylation (128). This is supported by the fact that the expression of MMP14 and CD44 is reduced and the migration of cells in basal breast cancer is reduced by knockdown of Ubc9 and PIAS1, which suggests that SUMOylation has a significant effect on the stability of CD44 (128, 129). At the same time, small molecule inhibitors of SUMO in primary colorectal cancers could inhibit the mRNA levels and protein expression of CD44, and the former decreased more significantly *in vitro* (130). However, Huang, X et al. proved that deficiency of Ubc9 could lead to an increase in CD44^+^ stem cells, which promoted the proliferation and migration of bladder cancer cells (131). These seemingly contradictory outcomes indicate that different mechanisms of SUMOylation regulating CD44 expression exist in different cell tumor microenvironments. A clearer insight of the molecular environment and the different cellular components that elicit differential results of CD44 will be essential to understand the effects of the SUMOylation modification on renewal in CSCs. A schematic representation of the crosstalk between SUMOylation and Wnt/β-catenin pathway is shown in **Figure 2**.

## Wnt/β-Catenin Signaling Regulates SUMOylation

Several studies have reported that Wnt/β-catenin pathway could regulate the SUMOylation of other proteins (132, 133). More recently, Satow et al. showed that Wnt proteins regulated SUMOylation of ZIC5 (132). Wnt activity could promote the binding of SUMO to lysine residues in the highly conserved (ZF-NC) domain of ZIC5, which was critical for neural ridge development in mice. Interestingly, after treating the HEK293T cells with the GSK-3β inhibitor LiCl, the proportion of SUMOylated ZIC5 increased, whereas the ability of ZIC5 to inhibit TCF-dependent transcription decreased in a time-dependent manner (132). This study reveals the importance of SUMOylation of the ZF-NC domain of ZIC5 and the activity of Wnt in neural crest development in mice. It has also observed that targeting global SUMOylation or Wnt may have potential side effects, and a more precise location and time of intervention of SUMOylation need to studied in detail. Furthermore, another study showed that β-catenin interacted with promyelocytic leukemia protein (PML) transcript variant IV and disrupted PML nuclear body (NB) formation by inhibiting SUMOylation of PML transcript variant IV mediated by RanBP2 (133). Furthermore, Picard et al. reported that a striking decrease of SUMOylated estrogen receptor (ER) β appeared after the inhibition of GSK-3β expression. The SUMOylation modification of ERβ regulated by GSK-3β was associated with the stabilization and transcriptional activity of ERβ (134). ERβ could interfere with ERα-mediated oncogenic proliferation of breast cancer cell (135).

## Conclusion

Genome-wide mapping techniques and proteomics have further promoted the study of dynamic changes in Wnt signaling networks and transcription complexes. These SUMO families have been found to effectively integrate into the Wnt signaling pathway, playing a key role in cancer self-renewal. Just as normal crosstalk between Wnt signaling and the SUMOylation modification may promote self-renewal of stem cells and organization, aberrant crosstalk may be tumorigenic in different systems. Finally, the influence of the SUMOylation modification on Wnt signaling appears to extend to CSCs, the most primitive cancer cells. As Wnt/β-catenin signaling seems to be required for stem cells and cancer cells, the ability of the SUMOylation modification to modulate Wnt signaling either positively or negatively may be of therapeutic relevance. Thus, understanding the interference of the SUMOylation modification on Wnt signaling may allow us to better control cancer cells when the treatment effect is unsatisfactory. SUMOylation on the key proteins involved in Wnt/β-catenin signaling pathway and their corresponding effects on the activity of Wnt/β-catenin signaling pathway have been showed in **Table 1**. Targeting SUMOylation modification of proteins has been proposed for the treatment of cancer. Several researchers have proposed improving the efficiency of cancer therapy by modifying targeted cancer pathways using SUMO, including small-molecule inhibitors. GA, a small-molecule SUMO inhibitor, could inhibit the growth of basal breast cancer xenografts in mice (128). Liu et al. demonstrated that GA could inhibit carcinogenesis and cancer progression in oral squamous cell carcinoma by inhibiting Smad4 SUMOylation in a time- and concentration-dependent manner (122). However, SUMOylation modification is also subject to a series of precise regulations, and has different binding forms with target proteins. In addition, tumor microenvironment can also regulate the SUMOylation modification to exert different functions. At present, most of the studies about SUMOylation modification were finished *in vitro and in vivo*, cell and animal experiments were difficult to simulate the microenvironment of human malignant tumors. Furthermore, while we focused on the interaction between SUMO and different Wnt members, the effects of SUMOylation modification on proteins other than Wnt signaling pathway cannot be ignored.

**Table 1:** SUMOylation on the key proteins involved in Wnt/β-catenin signaling pathway and their corresponding effects on the activity of Wnt/β-catenin signaling pathway.

Proteins	SUMOylation sites	E3	SUMOylation and deSUMOylation	Activity of Wnt signaling pathway	Cancers and cell lines	References
β-catenin	no available	RanBP2	SUMOylation	up	hepatocellular carcinoma	(67, 69, 70) (72, 73),
			deSUMOylation	down		
GSK-3β	Lys292	no available	SUMOylation	down	COS-1 cell line	(81)
			deSUMOylation	up		
TCF	Lys297	PIASy	SUMOylation	up	Hela S3 cell lines	(70)
			deSUMOylation	down		
LEF1	Lys 25 and Lys 267	PIASy	SUMOylation	down	Jurkat cell lines	(86)
Axin	six amino acids in the C-terminus	PIAS1, PIASx-β, and PIASy	SUMOylation	no significance	293T cell line	(89)
Axam	no available	no available	deSUMOylation	down	COS and SW480 cell lines	(94)
CBP	Lys999, 1034, 1057 and the ZZ domain	no available	SUMOylation	down	MACH-1, COS-1, and 293 cell lines	(98, 100)
			deSUMOylation	up		
CtBP1	Lys428	PIAS1, PIASx-β and Pc2	SUMOylation	up	HeLa and A549 cell lines	(103, 104) (105–107)
			deSUMOylation	down		
Smad3	C-terminal domain	PIASy	SUMOylation	up	COS, 293T and Hep3B cell line	(116–118)
Smad4	Lys159 and Lys113	PIAS1 PIASx-α, PIASx-β and PIASy	SUMOylation	up	colorectal cancer and oral squamous cell carcinoma	(120–122)
CD44	no available	PIAS1	SUMOylation	unknown	breast cancer, colorectal cancer, and bladder cancer	(128, 129)
			deSUMOylation			

Wnt/β-catenin pathway components undergo SUMOylation at different reaction levels. Owing to constantly evolving technology, the stage in which the SUMOylation modification may influence Wnt signaling in cancer cells may extend beyond the examples illustrated above. This pathway regulation is further complicated by the fact that the SUMO family is involved in many component modifications and is subject to crosstalk with other PTMs. SUMOylation of the same protein may result in opposite regulation of different downstream effector proteins. The same protein in cancer cells signals multiple dynamic changes in response to internal and external environmental changes in different cancer niches. The challenge for the future is that small-molecule inhibitors of SUMO in different stages of Wnt/β-catenin should be more targeted and spatio-temporally specific to achieve a precise impact on cancer stem cells under macro conditions.

## Author Contributions

SZ and MG designed the paper; contributed to manuscript writing; and approved the manuscript before submission. LF, XuY and MZ contributed to manuscript writing; and approved the manuscript before submission. XiY and YN and LF collected literatures and approved the manuscript before submission. All authors contributed to the article and approved the submitted version.

## Funding

This work was supported in part by grants from the National Science Foundation of China (#82173283 and #82103088), and Foundation of committee on science and technology of Tianjin (#21JCZDJC00230, #2020036, #20JCYBJC01230). The funders had no roles in the design of the study, data collection, analysis and interpretation, or decision to write and publish the work.

## Conflict of Interest

The authors declare that the research was conducted in the absence of any commercial or financial relationships that could be construed as a potential conflict of interest.

## Publisher’s Note

All claims expressed in this article are solely those of the authors and do not necessarily represent those of their affiliated organizations, or those of the publisher, the editors and the reviewers. Any product that may be evaluated in this article, or claim that may be made by its manufacturer, is not guaranteed or endorsed by the publisher.

## Abbreviations

PTM, post-translational modification; CSCs, cancer stem cells; LRP5/6,LDL receptor-associated protein 5/6; DVL, dishevelment; APC, adenomatous polyposis coli; GSK-3β, glycogen synthase kinase 3β; TCF,T-cell factor; LEF, lymphoid enhancer factor; SUMO, Small Ubiquitin-like Modifier; CBP, CREB binding protein; CtBP, C-terminal binding protein; SIM, SUMO interaction motif; Ubc9,ubiquitin conjugating enzyme; StUbls, SUMO-targeted ubiquitin ligase; SENP1,sentrin-specific protease 1; DeSI1, desulphurized isopeptidase 1; USPL1, ubiquitin-specific protease-like 1; NPC, the nuclear pore complex; GA, ginkgolic acid.
